# Patents and Patentees

**Published:** 1853-10

**Authors:** J. W. Keyes

**Affiliations:** Quincy, Fla.


### ARTICLE II.

*Patents and Patentees.* By J. W. KEYES, M. D., D. D. S., of Quincy, Fla.

IN the April number of the American Journal of Dental Science, we find an article by Dr. Hill, in which he proposes to "clear away the rubbish and extraneous matter which has accumulated about the subject of patents, and prove that a man has a legal, moral and professional right" to patent his inventions and improvements.

It appears to us, that in the following remarks, he has admitted all for which the advocates of the opposing side contend. He says, "when an individual is admitted to fellowship in any society having a constitution or by-laws, or both, it is expected of him that he will conform both to their letter and spirit. If one of these articles forbid the procurement of a patent, and he becomes a patentee, it is clear that he has violated the principles of fellowship in such society. His offence is a conventional one, and under certain circumstances may be characterized as an immorality.

"But if there is no article in the constitution or by-laws, or vote, or resolution passed by the society, which prohibits the right of any member to secure letters patent, has he then violated the terms of the compact? We think not.

"We will go farther and say, that if it can be shown that immemorial custom and usage in the society of which he is a member, has reprobated the principle of patents, he has committed an offence for which due atonement should be made. Can this be said of our profession, or any society of practitioners connected with it? If so we stand convicted, if not we are clear. With respect to the medical profession proper, we are aware of a strong feeling against the principle of patents in the profession, and we have been taught to regard it as *dishonorable* in a medical practitioner to become a patentee in things pertaining to practice, although the catalogue of M. D's guilty of this offence is really formidable."

Although there may be nothing in the constitution or by-laws, no resolution or vote of the American Association of Dental Surgeons against the principle of patenting in the profession, is not, we would ask, the *feeling* of the association against it? Does not our oldest and most distinguished schools disapprove of it? Is it not, at least, a doubtful propriety? Is not a large majority of the profession opposed to it? and does not this majority embrace a large proportion of those who have done *most* to elevate the profession to its present respectability and usefulness?

Has not immemorial custom and usage in the medical profession reprobated the principles of patents, and does not the *feeling* at the present day set *strongly* against it? Are there any among the fathers of medicine in this country, any of science's most distinguished sons, any of those who have done most to develop the resources of the profession, who do not reprobate this principle as improper and as tending to degrade the science of medicine? Do they not refuse to consult with the patentee? Is he, in any respect, admitted to terms of *equality?* Is not the hand of fellowship withheld, and is he not treated as an *outlaw?* Whence is the profession of dentistry? What its parentage? Is it not to all intents and purposes a part of medicine, a branch, a specialty merely? Can you mark out strictly where the trunk ceases or the branch begins? If then it is wrong in a physician to patent pills or practice, it must of consequence also be for the dentist.

Every profession has its infancy, its childhood, and its manhood. When in the infancy of dentistry, the practice was confined to barbers and those of like class, into whose hearts the idea of improvement, unconnected with profit, never entered, a species of patenting might have been expected, it was the natural result of the motive to improvement. Here the professional trade, would have rested, remaining in infancy. But men who looked beyond *this* and coupled with gain the general good, saw the necessities of society, and actuated by a desire to do good as well as get gold, adopted this as their vocation, *then* the infant began to grow, felt the genial influence and speedily expanded into maturing usefulness. Just in proportion as *this* class of men came into the profession, did its standard become elevated, its usefulness enhanced. And just here is the pivot upon which turns a feeling. It is this desire to do good, which acts as leaven to the loaf, which makes the difference between the profession and the trade. Will the encouragement of patenting elevate the profession in respectability or usefulness? Will it induce men of merit, of means, of education and talents to adopt it as their pursuit? On the contrary will not its encouragement tend rather to lessen its respectability? Suppose it was considered proper for a physician to patent pills and practice, how long before the profession would cease to exist as a calling, high and responsible? How long before the conscientious, honest, highminded, educated, would cease to enter it? How long before it would be a disgrace to be a *doctor* and ignorant patent-physic pretenders doling out their damning doses made up the mass of those dignified with the title? How long before it would be regarded with the same contempt, held in the same estimate which dentistry was a few years ago, when dentist and villanous vagrant were synonymous?

It is asked why not put the services of the physician and dentist upon the same footing as the clergy, viz. the voluntary contributions of the people, and thus make them free to the poor and abject portions of the community? We answer that there is where it should be, where, if the medical profession were properly purged and the people enlightened it would be, and where we hope at some day not far in the future it will be. Are not the services of the physician, and to some extent the dentist also, now free to the poor and abject? Does not the physician daily do charity, and the dentist too, to a less extent? The services of the dentist are not so much required as those of the physician, for the necessities of the poor and abject are not the same as those of people higher in life. The loss of a tooth to a man in one station of life is a *very* serious matter, whilst to another moving in a different sphere, it is a trifle. Has a dentist ever been known to turn away an applicant to have his tooth extracted because the applicant was unable to pay for the operation? Does the surgeon refuse to operate because the patient is poor, if the operation is necessary to relieve suffering or save life? We answer no, so far as we have ever known, emphatically no. Is this the case with the patentee? Take as an illustration Dr. Banning, "he has spent much time and money in perfecting a—contemptible—body brace." During this same period "Dr. Nameless" has spent much time and money in acquiring skill in surgery. Here then, without a patent they are upon an equal footing. Dr. B. receives high fees for body bracing, Dr. N. nothing for his operations. Dr. B. takes out a patent, and unless you brace his pocket, he will not brace your body, whilst Dr. N. will amputate your legs without charge, and dismiss you with his blessing and a part of his purse.

But "suppose the talents of some member of the dental profession should lead him into a series of experiments, which eventuates in the discovery of a process by which his professional brethren can be greatly advantaged and immense good to mankind result from it, meantime, in perfecting and bringing his discovery before the world his means are all exhausted, his family destitute and he greatly embarrassed with debt, and that debt incurred in prosecuting his experiments. How is he to be indemnified?"

Suppose after he has received high encomiums, gold medals, etc., his improvement is *worthless*—how is he to be indemnified? Suppose he patented it, sold privileges and rights, and it is of little or no value, what is to be done with him? Suppose he beggared his family in pursuit of some *great invention* and did not make it, how is he to be indemnified? Clearly, a man has no right to risk the well-being, the subsistence of his family upon the cast of a die. But this man has made an invention "of great advantage to his professional brethren and of immense good to mankind." Then let him rest for his reward upon the high position which it gives, the increased patronage which it brings. Let congress vote him a sum proportioned to the benefits his discovery brings mankind. Let academies reward him, and colleges vote him honors—and *solid* honors when they can. Let him not take out a patent—for though there exists no legislative enactments against it, a man may not do all the law allows.

It is said that "in all the high sounding verbiage, selfishness, like a serpent, lies concealed and demands the time, labor and money of the inventor without compensation." We ask what you have received? Nothing? Have you contributed so much that you must have *extra* pay? Are the various, valuable improvements and discoveries daily spread upon the pages of our journals of no value to you? Cost they, their authors, nothing?

Is the accumulated knowledge of all those who have gone before and cotemporary with you of no value? Every one contributes according to his means, and if you, by virtue of superior talents, are able to contribute much, it is, at least, no more than the widow's mite. We enter the brotherhood with the tacit understanding that we will do what we can to advance and improve it, and it behooves us to do whatever our hands may find to do. "Men's genius and talents are various and sure in different channels; on what principle is the labor of one man to be appropriated to the use of the public without reward and another extravagantly paid for his services?" Aye—abstractly—upon what principle? How is it that there is a difference between men? Upon what principle is it that this man toils, even unto death, and leaves his labors to bless the world, whilst another, who never advanced an idea, never relieved a want, rolls in affluence through a long life? Upon what principle is it that one man labors hard all day and receives one dollar, whilst another with little labor receives ten? Upon what principle is it that you, by the utmost tension of mental and physical powers for twelve months can earn only three or five thousand dollars, whilst Webster in a day, aye or a simple yea or nay, or even a nod of his great head, should receive ten thousand? Here are men of the same profession, who have labored harder than Webster ever did, and yet they barely subsist.

A parallel has been attempted between the author and the dentist, but, to our minds, there is an essential difference. The author rests for his remuneration upon securing his copyright, just as the dentist does upon his fees. If he invents a new kind of *verse*, for instance, he does not patent it, but relies upon the "*glory*" and the increased patronage for his remuneration. He has no other fee than such as his books bring. This then is his profession, and in securing his patent right he does not go out of it. If your profession is to *invent*, secure your inventions by patent, but if it is to practice dentistry or medicine, cling to *it*—do not *alloy* it.

But we must close these desultory remarks—We are pained that any thing in our former article should have wounded Dr.

Hill, for his talents and attainments merit our admiration, and he has it. He was specified, because he stood so prominently; not because there was anything *venomous* in our feelings towards him, for we look longingly forward to the day when his "*early teachings*" will have its legitimate effect, and bring him, and all others, to "eschew the patent as they would dishonesty." We may not have succeeded in making this matter *plainer* to others, but to our own mind, it is clear that patents are morally and professionally wrong, because, they tend to degrade the profession, and as a consequence lessen its usefulness, and open a wide field for the exercise of quackery and dishonesty.

*May* 10*th*, 1853.

